# Challenges in the Diagnosis of SARS-CoV-2 Infection in the Nervous System

**DOI:** 10.3390/v16081247

**Published:** 2024-08-03

**Authors:** Samya Jezine Da Silva, Mauro Jorge Cabral-Castro, Cássia Cristina Alves Gonçalves, Diana Mariani, Orlando Ferreira, Amílcar Tanuri, Marzia Puccioni-Sohler

**Affiliations:** 1 Programa de Pós-Graduação em Doenças Infecciosas e Parasitárias, Faculdade de Medicina, Universidade Federal do Rio de Janeiro, Rio de Janeiro 21941-853, Brazil; 2 Departamento de Patologia, Faculdade de Medicina, Universidade Federal Fluminense, Niterói 24220-900, Brazil; 3 Laboratório de Líquido Cefalorraquidiano, Hospital Universitário Clementino Fraga Filho, Universidade Federal do Rio de Janeiro, Rio de Janeiro 21941-913, Brazil; 4 Laboratório de Virologia Molecular, Departamento de Genética, Instituto de Biologia, Universidade Federal do Rio de Janeiro, Rio de Janeiro 21941-902, Brazil; 5 Departamento de Medicina Geral, Escola de Medicina e Cirurgia, Universidade Federal do Estado do Rio de Janeiro, Rio de Janeiro 22290-250, Brazil

**Keywords:** accuracy, neuroCOVID-19, cerebrospinal fluid, anti-SARS-CoV-2 immunoglobulins, inflammatory biomarkers, molecular biomarkers, viral infections, neuroinflammation

## Abstract

Neurological involvement has been widely reported in SARS-CoV-2 infection. However, viral identification in the cerebrospinal fluid (CSF) is rarely found. The aim of this study is to evaluate the accuracy of virological and immunological biomarkers in CSF for the diagnosis of neuroCOVID-19. We analyzed 69 CSF samples from patients with neurological manifestations: 14 with suspected/confirmed COVID-19, with 5 additional serial CSF samples (group A), and as a control, 50 non-COVID-19 cases (group B—26 with other neuroinflammatory diseases; group C—24 with non-inflammatory diseases). Real-time reverse-transcription polymerase chain reaction (real-time RT-PCR) was used to determine SARS-CoV-2, and specific IgG, IgM, neopterin, and protein 10 induced by gamma interferon (CXCL-10) were evaluated in the CSF samples. No samples were amplified for SARS-CoV-2 by real-time RT-PCR. The sensitivity levels of anti-SARS-CoV-2 IgG and IgM were 50% and 14.28%, respectively, with 100% specificity for both tests. CXCL-10 showed high sensitivity (95.83%) and specificity (95.83%) for detection of neuroinflammation. Serial CSF analysis showed an association between the neuroinflammatory biomarkers and outcome (death and hospital discharge) in two cases (meningoencephalitis and rhombencephalitis). The detection of SARS-CoV-2 RNA and specific immunoglobulins in the CSF can be used for neuroCOVID-19 confirmation. Additionally, CXCL-10 in the CSF may contribute to the diagnosis and monitoring of neuroCOVID-19.

## 1. Introduction

The severe acute respiratory syndrome coronavirus-2 (SARS-CoV-2) is an enveloped virus, and its genetic material consists of a single-stranded RNA (ribonucleic acid) molecule with positive polarity and an approximate size of 32 kilobases [1]. Its genome encodes proteins involved in the replication process and four structural proteins: the envelope protein (E), spike glycoprotein (S), membrane protein (M), and nucleocapsid protein (N) [1,2,3].

From December 2019 to July 2024, the World Health Organization (WHO) reported more than 775 million cases of COVID-19 worldwide and more than 7 million deaths. In Brazil, there were more than 37 million confirmed cases of COVID-19 and approximately 702,116 deaths [4].

SARS-CoV-2 infection is characterized by respiratory symptoms in the lower airways and a systemic inflammatory response, resulting in coronavirus disease 2019 (COVID-19) [5].

The neurological manifestations associated with SARS-CoV-2 infection have a frequency range between 50% and 82% [6,7], and include symptoms such as headache [8], as well as more serious complications such as encephalitis, myelitis, Guillain–Barré syndrome, peripheral neuropathies, and cerebrovascular diseases [9]. Long-term symptoms (symptoms that remain or new symptoms that occur after three months from the initial COVID-19 infection and may last for at least two months without another reason) can also occur, characterized by long COVID. In these situations, the consequences are serious, and the CSF can present a persistent inflammatory profile [10].

Infection in the nervous system may result from the virus’s direct action and/or by many indirect action mechanisms. SARS-CoV-2 has an affinity for cellular receptor ACE-2 (angiotensin-converting enzyme-2), which is widely distributed in cells of the nervous system (astrocytes, neurons, oligodendrocytes, olfactory bulb); this affinity allows the virus to pass directly through the blood–brain and blood–CSF barriers. However, neurological manifestations can also be caused by indirect effects of the virus, such as hypoxia caused by pulmonary infection, which can cause intracranial pressure and acute ischemic stroke, or immune damage caused by the excessive production of pro-inflammatory cytokines, called a “cytokine storm”. The affinity for the ACE receptor can also change blood pressure, which can result in brain hemorrhage, and the high production of coagulation substances (d-dimer and antiphospholipid antibodies) can increase the probability of cerebrovascular disease [11].

Investigations into the neurological diagnosis of COVID-19 consist of the clinical evaluation of the patient, imaging examination through MRI (magnetic resonance imaging) or CT (computed tomography), and CSF analysis [10]. Despite the high frequency of neurological symptoms alongside SARS-CoV-2 infection, neuroCOVID-19 may be underdiagnosed due to the low detection of SARS-CoV-2 in the CSF, mainly in cases where indirect action by the virus occurs. In this sense, this study aims to evaluate the accuracy of specific immunoglobulins and other neuroinflammatory biomarkers (neopterin and CXCL-10) in cerebrospinal fluid (CSF) samples for the diagnosis of neuroCOVID-19.

## 2. Materials and Methods

### 2.1. Patient Samples

This is a cross-sectional observational study. A total of 69 CSF samples were collected from patients with neurological disorders and divided as follows: Group A—14 CSF samples from 14 patients (confirmed/suspected COVID-19) during the pandemic period, with an additional 5 serial examinations from second and third CSF lumbar punctures. As the control, 50 CSF non-COVID-19 samples from the pre-pandemic period were used. Group B included 26 CSF samples from 26 patients with neuroinflammatory diseases with inflammatory CSF (cell count > 4 cells/mm^3^, protein > 45 mg/dL), and group C included 24 CSF samples from 24 patients with non-neuroinflammatory diseases with normal routine CSF. The coronavirus disease 2019 (COVID-19) case definition was based on the Brazilian Ministry of Health criteria [12].

### 2.2. Routine Cerebrospinal Fluid Analysis

The CSF samples were analyzed for cell count, protein and glucose concentration, bacteria, fungi and mycobacteria, and VDRL.

### 2.3. Real-Time RT-PCR for SARS-CoV-2 RNA in CSF Samples

For SARS-CoV-2 detection by real-time RT-PCR, specific primers and probes were used for the nucleocapsid regions (N1 and N2) and the endogenous control (RNaseP) [13], using the commercial GoTaq Probe One-Step RTqPCR kit (Promega, Madison, WI, USA).

### 2.4. Anti-SARS-CoV-2 IgM and IgG antibodies in CSF Samples

The research on anti-SARS-CoV-2 IgM and IgG antibodies was carried out using the commercial kits BIOLISA SARS-CoV-2 IgM (Bioclin, Belo Horizonte, Brazil) and Anti-SARS-CoV-2 ELISA IgG (EUROIMMUN, Lübeck, Germany).

### 2.5. Neopterin and CXCL-10 in CSF Samples

The concentrations of neopterin and CXCL-10 in the CSF samples were determined using the commercial kits Neopterin ELISA (IBL International GMBH, Hamburg, Germany) and IP-10 (CXCL-10) Human ELISA (Invitrogen, Carlsbad, CA, USA). All of the commercial tests followed the manufacturer’s recommendations, with one adaptation for 1:2 dilution in the CSF samples, per a previous validation for specific immunoglobulin ELISA tests.

### 2.6. Statistical Analysis

GraphPad Prism version 8 (GraphPad Software, La Jolla, CA, USA) was used, assuming *p* < 0.05 as the significance level. Categorical data are represented as the median, minimum, and maximum. The Kruskal–Wallis test was used to assess the significance between the three different groups, and Dunn’s test was used for multiple comparisons. Receiver operating characteristic (ROC) curve analysis was used to obtain the best cut-off value for neopterin and CXCL-10, and the accuracy of the molecular and IgG/IgM anti-SARS-CoV-2 tests was evaluated for sensitivity, specificity, and positive and negative predictive values (PPV and NPV), expressed in percentages (%).

## 3. Results

### 3.1. Patient Characteristics

Of the fourteen patients in group A (confirmed/suspected COVID-19), eight were classified as suspected cases of COVID-19 (six with influenza-like illness (ILI) and two with severe acute respiratory syndrome (SARS)), and six patients were classified as confirmed cases of COVID-19. Five of them fulfilled the clinical laboratory criteria, based on a positive result in the real-time RT-PCR with nasopharyngeal swab samples, and another fulfilled the clinical imaging criteria based on CT compatible with COVID-19 (50% lung involvement) and dyspnea (Table 1). The neurological manifestations of the 15 COVID-19 cases included encephalitis (2), meningoencephalitis (4), encephalopathies (6), rhombencephalitis (1), and meningitis (1) (Table 1).

The 14 patients from group A had a median (min–max) age of 62.5 (19–79) years and were predominantly male (71.43%). Eight out of fourteen (57.14%) progressed to death, and the median (minimum and maximum) time from onset of COVID-19 symptoms to the first lumbar puncture (LP) was 15.5 days (1–55). The clinical characteristics and CSF findings are shown in Table 1. Seven out of eight (14 87.5%) patients with suspected COVID-19 presented brain involvement, and 62.5% (5/8) died. Those with confirmed COVID-19 presented brain involvement in 100% (6/6) of cases, and 50% (3/6) died. The CSF analysis of the confirmed and suspected cases were also similar ). All CSF samples from group A had a negative microbiological analysis.

**Figure 1:**
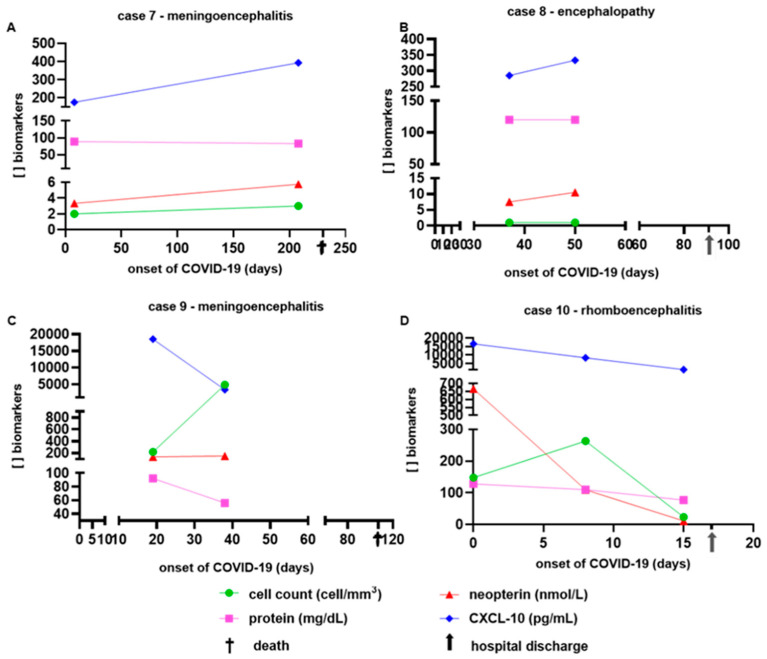
Demonstration of the concentration of inflammatory markers (cell count, protein, neopterin, and CXCL-10) in four cases of neuroCOVID-19 with serial CSF study. The meningoencephalitis case in graph (**A**) presents a progressive increase in inflammatory markers of cell count, neopterin, and CXCL-10 as the days passed until the patient died, while case 10 (rhombencephalitis) in graph (**D**) demonstrates a sudden drop in these same markers until day 15, when the patient was discharged from hospital. In the same graphs (**B**,**C**), we can observe case 8 with encephalopathy (hospital discharge) and case 9 with meningoencephalitis (death). A more detailed analysis was not possible in these cases as the last lumbar puncture performed occurred long before these cases presented an outcome.

The serial analysis of the markers (cell count, protein, neopterin, and CXCL-10) in the CSF samples (second and third PL) from four cases from the COVID-19 group are shown in Figure 1. Case 7, with meningoencephalitis (A), presented a progressive increase in neuroinflammatory biomarkers (cell count, neopterin, and CXCL- 10 in CSF) until he died, while case 10, with rhombencephalitis (D), demonstrated a sudden drop in the same biomarkers until day 15, when the patient was discharged from hospital. In the same figure, via graphs B and C, we can observe case 8 with encephalopathy (hospital discharge) and case 9 with meningoencephalitis (death). A more detailed analysis was not possible in these cases as the last lumbar puncture performed occurred long before these cases’ outcomes.

The samples of groups B and C were from the pre-pandemic period. Group B (inflammatory control without COVID-19) was composed of a panel of 26 CSF samples collected from patients with other inflammatory neurological diseases: viral encephalitis (excluded for SARS-CoV-2) (*n* = 6); bacterial and fungal meningitis (*n* = 11); neurosyphilis (*n* = 1); myeloradiculitis (*n* = 1); metabolic encephalopathy (*n* = 2); and autoimmune diseases (*n* = 5). This group was 61.5% (16/26) male. In group C (non-inflammatory control group), 24 CSF samples collected from patients with the following neurological manifestations of non-inflammatory origin were selected: dementia (*n* = 4), neoplasm (*n* = 7), psychiatric disturbances (major depression and somatoform conversion) (*n* = 2), idiopathic intracranial hypertension (*n* = 3), metabolic disturbances (*n* = 6), microangiopathy (*n* = 1), and degenerative myelopathy (*n* = 1). The profile of patients in group C showed a predominance of females at 79.2% (19/24).

### 3.2. CSF Analysis

**Figure 2:**
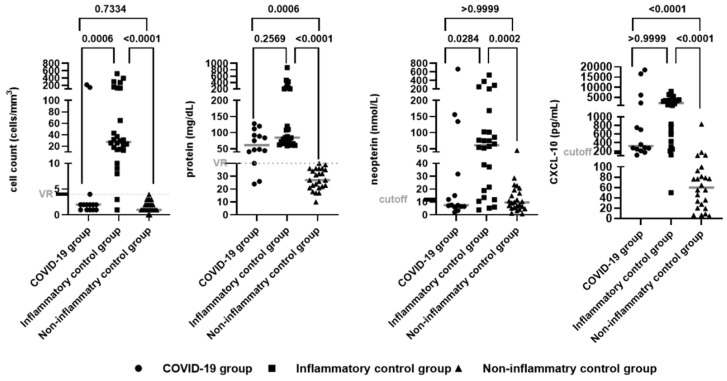
CSF analysis (cell count, protein concentration, neopterin, and CXCL-10) for the different groups of patients (neuroCOVID-19, inflammatory control group, non-inflammatory control group). The cell counts, proteins, and glucose measures are expressed as median values (minimum–maximum). The statistical significance of the CSF profile data was assessed between the groups (A × B, A × C, B × C) using the Kruskal–Wallis test and Dunn’s test of multiple comparisons. Reference values: cell count ≤ 4 cells/mm^3^, protein 15–40 mg/dL, glucose 45–70 mg/dL, cut-off of 11.87 nmol/L for neopterin and 174.7 pg/mL for CXCL-10, defined only for the neuroCOVID-19 group according to the ROC curve. For statistical significance, *p* < 0.05.

The median cell counts and neopterin concentrations in the CSF samples showed no difference between the groups of patients with neuroCOVID-19 and the non-inflammatory control group (*p* > 0.05). However, the medians of the proteins and CXCL-10 concentrations in the CSF samples were elevated in the group of patients with neuroCOVID-19 (group A) and the inflammatory control group (group B) when compared to the non-inflammatory control group (group C) (*p* < 0.05). The concentration of glucose in the CSF had no difference between the three groups analyzed (*p* > 0.05) (Figure 2).

In the accuracy evaluation of the molecular and immunological tests, we found in the COVID-19 group (A) that the sensitivity of IgG anti-SARS-CoV-2 in the CSF samples was 57.14% (8/14), which decreased to 50% (5/10) when CSF samples from patients previously vaccinated for COVID-19 were not considered. The positive and negative predictive values of the test for anti-SARS-CoV-2 IgG were 100% and 81.25%, respectively. The sensitivity for anti-SARS-CoV-2 IgM was 14.28% (2/14) for the neuroCOVID-19 group. The positive and negative predictive values of the test for anti-SARS-CoV-2 IgM were 100% and 81.25%, respectively. All CSF samples in the control groups (B and C) were non-reactive for IgG and IgM, showing 100% specificity. No samples were PCR-positive for SARS-CoV-2 in group A. The details are reported in Table 2.

**Figure 3:**
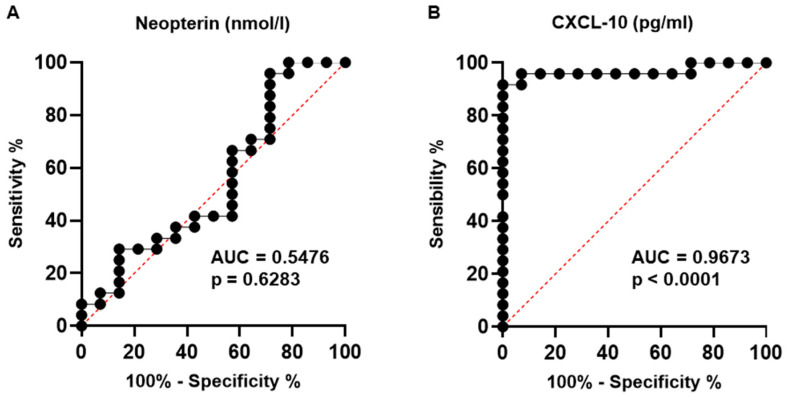
Receiver operator characteristic (ROC) curves constructed for neopterin (**A**) and CXCL-10 (**B**) in CSF samples from the COVID-19 group compared to the non-inflammatory control group. In both graphs, the black line represents the ROC curve and the red dotted line represents chance level performance. The neopterin test on CSF samples presented a failed performance, with an area under the curve (AUC) of 0.5476 (95% confidence interval [CI) 0.3470–0.7482; *p* = 0.6283]. A cut-off value of 11.87 nmol/L was determined, presenting a sensitivity of 66.67% and specificity of 42.86%. The CXCL-10 test presented the best overall performance, with an AUC of 0.9673 (95% confidence interval [CI) 0.9078–1.000; *p* < 0.0001], a cut-off of 174.7 pg/mL, and sensitivity and specificity values of 95.83% and 92.86%, respectively.

Using ROC (receiver operator characteristic) curves, we evaluated the neuroinflammatory biomarkers of neopterin and CXCL-10 in the CSF samples to differentiate the group of neuroCOVID-19 (group A) from the non-inflammatory control group (group C). The neopterin test on the CSF samples presented a failed performance, with an area under the curve (AUC) of 0.5476 (95% confidence interval [CI) 0.3470–0.7482; *p* = 0.6283]. A cut-off value of 11.87 nmol/L was determined, presenting a sensitivity of 66.67% and specificity of 42.86%. The CXCL-10 test had the best overall performance, with an AUC of 0.9673 (95% confidence interval [CI) 0.9078–1.000; *p* < 0.0001], a cut-off of 174.7 pg/mL, and sensitivity and specificity values of 95.83% and 92.86%, respectively (Figure 3).

**Figure 4:**
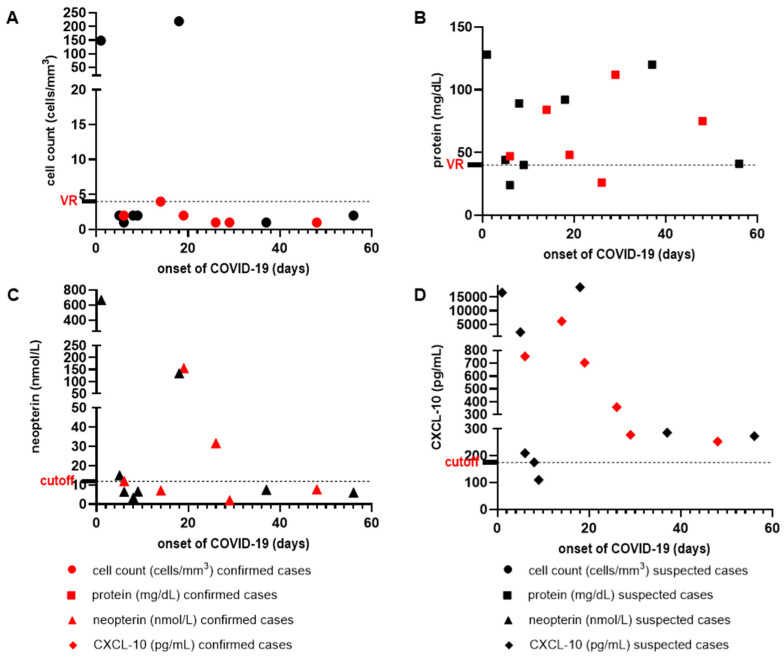
Distribution of the values found for inflammatory markers (cell count, protein concentrations, neopterin, and CXCL-10) in relation to the onset of COVID-19 symptoms (days) among cases of suspected COVID-19 (in black) and confirmed cases of COVID-19 (in red). (**A**)—distribution of cell counts values (cells/mm^3^) vs. time of COVID-19 symptoms; (**B**)—distribution of protein concentration values (mg/dL) vs. time of COVID-19 symptoms; (**C**)—distribution of neopterin concentration values (nmol/L) vs. time of COVID-19 symptoms; (**D**)—distribution of CXCL-10 concentration values (pg/mL) vs. time of COVID-19 symptoms.

We also demonstrated the distribution of the values found for neuroinflammatory biomarkers (cell count, protein concentrations, neopterin, and CXCL-10 in the CSF) with regard to the duration of COVID-19 symptoms in days. We observed no difference among suspected and confirmed cases in the neuroCOVID-19 group. Only two situations (case 9 with meningoencephalitis and case 10 with rhombencephalitis in graph A) presented a cell count lower higher than the reference value (>4 cells/mm^3^), and only one (case 14 with encephalitis in graph D) presented a CXCL-10 concentration below the cut-off defined by the ROC curve (<174.7 pg/mL). These concentrations in relation to the duration of COVID-19 symptoms are shown in Figure 4.

## 4. Discussion

The diagnosis of SARS-CoV-2 infection in the nervous system is a challenge since the detection of the virus is rarely found in CSF. In general, the sensitivity of SARS-CoV-2 RT-PCR in respiratory samples is between 68 and 75% [14]. Some improved molecular methods (RT-LAMP with simultaneous use of two primers sets—ORF1a-HMS/Gene E) have achieved a better sensitivity of 81.8% with nasopharyngeal swab samples [15], but in CSF samples the sensitivity is always low, at around 6 to 9% [16,17]. This means that some patients with neuroCOVID-19 may go undiagnosed.

There are different circumstances reported that could lead to a negative detection of SARS-CoV-2 in CSF samples by RT-PCR, such as using methods based on different protocols or even case report studies, low amounts of viral particles in the CSF, and/or a loss of the virus detection period by the time of the test. Furthermore, the neuropathogenesis of COVID-19 may be associated with other mechanisms, such as metabolic alterations caused by cytokine storms, hypoxia associated with pulmonary infections leading to ischemic stroke, hypertensive pressure resulting in brain hemorrhage, or autoimmune diseases [9,16,18,19]. These mechanisms of indirect damage, as well as different protocols of methods and laboratory analyses, could make the diagnostic confirmation of neurological diseases caused by SARS-CoV-2 difficult.

In this study, no CSF samples were amplified for SARS-CoV-2 by real-time RT-PCR. Although molecular testing is the reference standard for viral diagnosis in the nervous system, specific antibodies are used to support other neurological infectious conditions, such as arboviruses, neurosyphilis, and neurocysticercosis [20,21,22]. For this reason, the negative detection of SARS-CoV-2 in CSF and respiratory samples does not exclude neuroCOVID-19. Based on these arguments, we used the clinical diagnosis of neuroCOVID-19 as an imperfect reference standard [23] to evaluate the accuracy of immunological tests.

We found sensitivities of 50% and 14% for the detection of anti-SARS-CoV-2 IgG and IgM, respectively, and a specificity of 100% for both tests in the neuroCOVID-19 group. Among the reactive CSF samples for anti-SARS-CoV-2 IgG, two CSF samples were from suspected/unconfirmed cases of neuroCOVID-19 (one encephalopathy case and one rhombencephalitis case) without previous vaccination, establishing that cases of neuroCOVID-19 are underdiagnosed by RT-PCR.

A systematic review found that 6% (17/304) of CSF samples were positive for SARS-CoV-2 and 12% (7/58) were reactive for specific antibodies, with evidence of intrathecal synthesis [15]. Another prospective and multicenter study found that 44% (15/34) of CSF samples were reactive for IgG against the virus and no CSF samples were positive via RT-PCR [24]. Nonetheless, these studies used positivity for SARS-CoV-2 via RT-PCR or serological tests as a reference standard for their inclusion criteria.

Therefore, the question remains regarding underdiagnosed cases of neuroCOVID-19, as even without confirmation of the disease (i.e., suspected cases), patients could still present an immunological response in the nervous system detected by reactions to specific immunoglobulins in the CSF.

In addition, we demonstrated high CSF protein and CXCL-10 concentrations in the neuroCOVID-19 group, showing significant differences in relation to group C (non-inflammatory). CXCL-10 presented the best performance, determined by its ROC curve, as a neuroinflammatory biomarker in comparison to the other biomarkers. We found a cut-off of 174.7 pg/mL and sensitivity and specificity values of 95.83% and 92.86%, respectively.

In a previous study of biomarkers in dengue and chikungunya neuroinvasive conditions, CXCL-10 in the CSF presented a 156.5 pg/mL cut-off with 91.7% sensitivity and specificity for the diagnosis of these conditions [25]. This neuroinflammatory biomarker may be produced by neurons, glial cells, microglia, or astrocytes. Therefore, they can also be analyzed as a possible tool for identifying inflammatory central nervous system damage by SARS-CoV-2. Some studies have already suggested the association of microglia damage and the release of cytokines (interleukin-6, IL-8, IL-10, CXCL-10, TNF-α, and Neopterin) in serum and CSF samples in SARS-CoV-2 infection [26,27].

The concentration of neopterin and CXCL-10 is high in the nervous system due to the intrathecal production of these markers, even under normal conditions [25]. Therefore, the evaluation of neopterin and CXCL-10 can contribute to diagnosis in cases of neuroinfections where the virus is not detected in CSF samples.

In a cohort study with 35 neuroCOVID-19 patients, elevated levels of interleukin-6 (IL-6) in the CSF were correlated with COVID-19 severity, [28]. Another study found elevated levels of CXCL-10 in CSF samples collected from patients who had inflammatory neurological diseases caused by COVID-19 (EDA, encephalitis, meningitis, meningoencephalitis, myelitis, and neuromyelitis optica) compared to a control group of patients who had non-communicable neurological diseases (infectious and non-inflammatory) from the pre-pandemic period (*p* < 0.01) [29].

In the present study, the detection of anti-SARS-CoV-2 IgG/IgM showed better accuracy than the molecular analysis of CSF. Moreover, two cases of suspected neuroCOVID-19 also had specific immunoglobulin in their CSF. The CXCL-10 marker presented excellent sensitivity and specificity, and was able to confirm the presence of neuroinflammatory reaction also in patients with suspected neuroCOVID-19.

It is important to evaluate the accuracy of new biomarkers for the diagnosis of neuroCOVID-19. Another promising protein is the activity-regulated cytoskeleton-associated protein (Arc protein). This protein plays an important role in synaptic plasticity in dendrites and in the molecular processes associated with learning and memory, more precisely, in regions such as the hippocampus and the cerebral cortex [30,31]. Some studies have related large amounts of Arc protein in neurons and astrocytes to diseases such as Alzheimer’s, neuroinfection, and even stroke occurring due to aneurysmal subarachnoid hemorrhage (SAH) [30,32,33,34]. It serves as an indicator of brain damage. For example, enhancements in the expression of Arc protein were observed in neuronal cells infected with HSV-1. This protein may contribute to the prognosis of neuroinflammatory and neuroinfection diseases [33].

Through the findings of our study, we have demonstrated that the neurological complications associated with COVID-19 are challenging to diagnose considering the low sensitivity of molecular and immunological tests. This condition may be underreported. For that reason, the absence of SARS-CoV-2 RNA and anti-SARS-CoV-2 IgG and IgM in CSF does not exclude the diagnosis, but rather their detection is confirmatory. Additionally, CXCL-10 concentration was found to be a potential biomarker of neuroinflammatory reaction in the CSF samples from patients with neurological disorders associated with COVID-19.

Since there is no standardized protocol for the diagnosis of neuroCOVID-19, a set of imaging analyses, CSF and serum tests, and clinical evaluation are necessary. In addition, the evaluation of new markers can bring new perspectives for the assessment of the neurological conditions presented in COVID-19, which can be acute, post-infectious, or even long-term manifestations, as is the case with long COVID.

The emergence of SARS-CoV-2 raises new questions in neurology, considering that the virus may be associated with frequent neurological conditions of high mortality and morbidity. The laboratory confirmation of neuroCOVID-19 diagnosis presents limitations due to the low sensitivity of specific immunological and molecular tests with CSF. On the other hand, positive specific tests confirm the diagnosis due to their high specificity. The underlying neuroinflammation in COVID-19 can be identified by detecting elevated levels of CXCL-10 in the CSF. 

The authors suggest that neuroCOVID-19 should be investigated in the diagnosis of cases of encephalitis/encephalopathy, meningitis, and myelitis of unknown etiology, especially if preceded by acute respiratory infection. Undiagnosed cases may represent a serious public health problem worldwide.

## Figures and Tables

**Table 1:** Characteristics of CSF samples from suspected and confirmed COVID-19 cases.

Patient (Sample)	COVID-19	Criteria Diagnosis	Duration of COVID-19 Symptoms until LP (Days)	Neurological Diagnosis	Neurological Manifestations	Vaccination	Cell Count in CSF (cells/mm^3^)	Red Cells in CSF (cells/mm^3^)	Protein in CSF (mg/dL)	Neopterin in CSF (nmol/L)	CXCL-10 in CSF (pg/mL)	IgG Anti- SARS-CoV-2 in CSF	IgM Anti- SARS-CoV-2 in CSF	Outcome
1	Confirmed	Clinical laboratory	13	Encephalopathy	Decreased level of consciousness	No	4	**7**	**84**	7.184	6189.299	Non- reactive	Non- reactive	Discharge
2	Confirmed	Clinical laboratory	18	Encephalopathy	Speech disorder, difficulty walking, disorientation	No	2	**778**	**48**	156.094	703.352	Reactive	Reactive	Death
3	Confirmed	Clinical laboratory	28	Encephalitis	Tonic-clonic seizure, decreased level of consciousness	No	1	**1**	**112**	2.089	277.206	Reactive	Non- reactive	Discharge
4	Confirmed	Clinical laboratory	25	Encephalopathy	Right hemiparesis	No	1	2	26	31.689	358.320	Non- reactive	Non- reactive	Discharge
5	Confirmed	Clinical laboratory	47	Encephalopathy	Decreased level of consciousness and convulsive crisis	No	1	**15**	**75**	7.736	252.834	Reactive	Reactive	Death
6	Confirmed	Clinical imaging	5	Meningoencephalitis	Decreased level of consciousness and convulsive crisis, MRI with focal lesion	Yes	2	**27**	**47**	12.00	752.266	Reactive	Non- reactive	Death
7 *	Suspected	SARS	7	Meningoencephalitis	disorientation, general decline	No	2	**0**	**89**	3.351	175.29	Non- reactive	Non- reactive	Death
8 *	Suspected	SARS	36	Encephalopathy	Changing the mental state	No	1	**1**	**120**	7.533	285.356	Non- reactive	Non- reactive	Discharge
9 *	Suspected	ILI	18	Meningoencephalitis	Decreased level of consciousness	No	**219**	816	**92**	134.770	18,533.200	Non- reactive	Non- reactive	Death
10 *	Suspected	ILI	1	Rhombencephalitis	Mental confusion (disorientation), ataxia, diplopia	No	**148**	**1**	**128**	666.860	16,589.611	Reactive	Non- reactive	Discharge
11	Suspected	ILI	4	Encephalopathy	Decreased level of consciousness	No	2	1	44	15.003	2253.635	Reactive	Non- reactive	Death
12	Suspected	ILI	5	Meningoencephalitis	Neck stiffness, change in level of consciousness	Yes	1	3	24	6.503	208.996	Reactive	Non- reactive	Death
13	Suspected	ILI	55	Meningitis	Change in level of consciousness, delirium, focal seizure	Yes	2	**81**	**41**	6.058	272.508	Non- reactive	Non- reactive	Death
14	Suspected	ILI	8	Encephalitis	Seizure	Yes	2	108	40	6.623	109.894	Reactive	Non- reactive	Discharge

Of the fourteen cases of neuroCOVID-19 in group A, eight were classified as suspected cases of COVID-19 because they had ILI (*n* = 6) and SARS (*n* = 2), and six patients were classified as confirmed cases of COVID-19. Five were confirmed by clinical laboratory criteria, i.e., presenting a positive result in the real-time RT-PCR with nasopharyngeal swab samples, and one case was confirmed by clinical imaging criteria, as they presented meningoencephalitis with compatible CT with COVID-19, showing 50% lung involvement and dyspnea. Reference values: leukocytes 4 cells/mm^3^, protein 15–40 mg/dL, glucose 45–70 mg/dL. Results in bold for CSF cell count, CSF red blood cells, and CSF protein concentration represent values outside the reference range. * Cases 7, 8 and 9 had a 2nd CSF sample as a control and case 10 had 2nd and 3rd CSF samples as controls. According to the Brazilian Ministry of Health, the cases were divided into suspected cases of COVID-19 (influenza-like illness (ILI) with at least two signs or symptoms (fever, sore throat, cough, headache, coryza, olfactory or taste disorders, chills) or severe acute respiratory syndrome (SARS)) and confirmed cases of COVID-19, defined as meeting (I) clinical criteria with the presence of ILI or SARS associated with anosmia or ageusia; (II) clinical epidemiological criteria with ILI or SARS (up to 14 days after contact with COVID-19); (III) clinical imaging criteria with ILI or SARS and computed tomography (CT) of the lungs characterized by COVID-19; (IV) laboratory criteria with a positive result in the molecular test (RT-PCR or RT-LAMP), or reactive results for IgM, IgG, IgA, anti-SARS-COV-2, or antigen detection by immunochromatography test in non-vaccinated patients; (V) laboratory criteria with a positive result in the molecular test or antigen detection for vaccinated patients; or (VI) laboratory criteria with a positive result in the molecular test or antigen detection for asymptomatic cases.

**Table 2:** Accuracy results of the tests for the different groups analyzed.

Tests	Accuracy Analysis	COVID-19
IgG anti-SARS-CoV-2	Sensitivity %	57% (8/14)
	Sensitivity %	50% (5/10)
	(Unvaccinated patients)	100%
	PPV %	81.5%
IgM anti-SARS-CoV-2	Sensitivity %	14.28% (2/14)
	PPV %	100%
	NPV %	81.5%

Sensitivity, specificity, PPV, and NPV are expressed in %. PPV—positive predictive value; NPV—negative predictive value.

## Data Availability

The data collected and analyzed were obtained according to the study methodology and used for scientific research only after approval by the HUCFF ethics and research committee, respecting the confidentiality of patients’ personal data. For more information, the authors should be contacted.
